# Construction of Multicolor Upconversion Nanotheranostic Agent for *in-situ* Cooperative Photodynamic Therapy for Deep-Seated Malignant Tumors

**DOI:** 10.3389/fchem.2020.00052

**Published:** 2020-02-11

**Authors:** Tongtong Hong, Yanxialei Jiang, Zihong Yue, Xinyue Song, Zonghua Wang, Shusheng Zhang

**Affiliations:** ^1^Shandong Sino-Japanese Center for Collaborative Research of Carbon Nanomaterials, College of Chemistry and Chemical Engineering, Qingdao University, Qingdao, China; ^2^Collaborative Innovation Center of Tumor Marker Detection Technology, Equipment and Diagnosis-Therapy Integration in Universities of Shandong, Shandong Provincial Key Laboratory of Detection Technology for Tumor Markers, College of Chemistry and Chemical Engineering, Linyi University, Linyi, China

**Keywords:** nanotheranostic agent, photodynamic therapy, reactive oxygen species, tumors, upconversion nanoparticles

## Abstract

Upconversion nanoparticles (UCNPs)-based photodynamic nanotheranostic agents could address the main drawbacks of photosensitizer molecules (PSs) including instability in aqueous solution and rapid clearance. Due to the relatively weak luminescence intensity of UCNPs and insufficient reactive oxygen species (ROSs), UCNPs-based photodynamic therapy (UCNPs-PDT) was discounted for deep-seated tumors. Thus, we proposed a PSs-modulated sensitizing switch strategy. Indocyanine green (ICG) as an NIR organic dye was proved to effectively enhance the luminescence intensity of UCNPs. Herein, four-color UCNPs were coated with a silica layer which loaded ICG and PSs while the thickness of silica layer was controlled to assist the sensitization function of ICG and activation of PSs. Under the drive of mitochondria-targeting ligand, the prepared nanotheranostic agent would accumulate in the mitochondria where ROSs were *in-situ* produced and then cell apoptosis was induced. Due to the cooperative PDT and high tissue-penetration depth of NIR laser, the prepared upconversion nanotheranostic agent could achieve significant inhibition on the deep-seated tumors.

## Introduction

As an exogenous stimulus for activatable theranostics, light presents the advantages of high spatiotemporal selectivity and negligible side effects, and has been widely applied for photothermal, photodynamic, and/or photo-triggered chemo/gene therapy (Huang et al., 2014). Photodynamic therapy (PDT) use photosensitizers (PS) to produce reactive oxygen species (ROSs) which could selectively and irreversibly destroy cancer cells and tumor tissue without damaging adjacent healthy ones. However, its clinical applications are mainly limited by its short tissue penetration depth, easily aggregated PS molecules and insufficient generation of ROSs (Chatterjee et al., 2018). Under the excitation of near infrared (NIR) light, upconversion nanoparticles (UCNPs) could emit Uv-Vis luminescence. Thus, UCNPs have the obvious merits of high tissue-penetration depth, negligible auto-fluorescence background, and low biotoxicity (Liu Y. et al., 2016). Under the excitation of NIR, UCNPs as the energy donor could effectively excite PS molecules (energy acceptor) to perform PDT via the luminescence resonance energy transfer (LRET) strategy (Fan et al., 2014; Liu et al., 2015; Chen et al., 2017). Nevertheless, the quantum yield and luminescence efficiency of UCNPs are relatively low due to the forbidden 4f-4f electronic transitions of lanthanide ions, weak absorption ability for NIR light and low doped concentration of the activators (below 2 mol%) (Ge et al., 2017). Thus, UCNPs-based PDT efficacy is discounted, especially for the deep-seated malignant tumors.

**Scheme 1:**
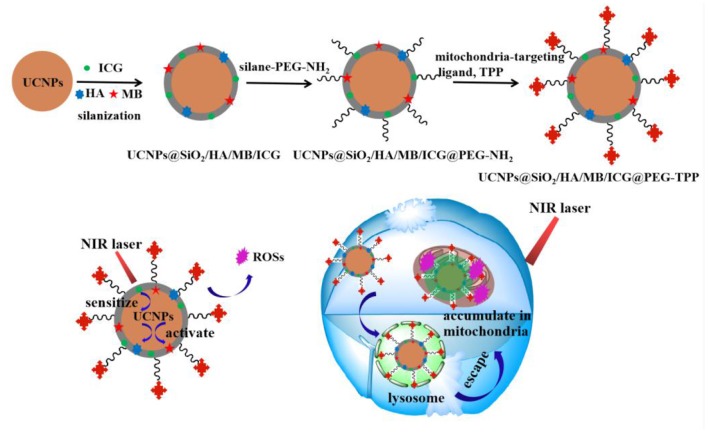
Schematic image for the synthetic procedure of the UCNPs@SiO_2_/HA/MB/ICG@PEG-TPP nanotheranostic agent and its theranostic functions for the photodynamic therapy.

Recently, an interesting dye-sensitization strategy was proposed to improve the absorption ability of UCNPs for NIR and enhance their UCL intensity (Wei et al., 2006; Zou et al., 2012, 2016; Chen et al., 2015; Wu et al., 2016; Lee et al., 2017; Wang et al., 2017; Xu et al., 2017; Garfield et al., 2018). In this sensitization system, organic dyes with strong NIR absorption ability could harvest NIR energy, then transfer its excited-state energy to sensitizer ions of UCNPs via multistep non-radiative energy transfer process. Based on its sensitization ability, a specific NIR organic dye was used as both the recognition unit for targets and an effective sensitizer for UCL to develop a target-modulated sensitizing switch to break through the signal-to-background limit of upconversion nanoprobes. The reaction between the dye and targets would switch on the sensitization and afford a significantly increased LERT efficiency (Liang et al., 2018). Herein, we tried to design a photosensitizers-modulated sensitizing switch for UCNPs-based photodynamic nanotheranostic agent. Among NIR organic dyes, indocyanine green (ICG) could effectively sensitize Yb^3+^ and largely improve the luminescence intensity of UCNPs via the Förster-type energy transfer (Yan et al., 2016). Approved by the U.S. Food and Drug Administration (FDA), and ICG could serve as an NIR agent to achieve photothermal therapy (PTT)/PDT effect under the excitation of 800 nm. As reported, UCNPs-based drug delivery systems have been developed to solve the intrinsic problems of ICG, including instability in aqueous solution, rapid clearance, self-bleaching as well as absence of targeting (Yan et al., 2016), and mainly applicated for PTT (Zheng et al., 2013, Huang et al., 2014; Lv et al., 2017). Moreover, UCNPs with four colors have not been studied for phototherapy. Herein, we developed a feasible cooperative PDT nanotheranostic agent which used four-color UCNPs as the core, a thin silica layer for loading triple photosensitizer molecules [Hypocrellin A (HA), methylene blue (MB) and ICG] as the medium layer and mitochondria targeting ligand modified polyethylene glycol as the outer layer. Under the excitation of NIR, UCNPs would excite ICG molecules, which would further transfer their excited energy to emitters ions, thus the luminescence efficiency of UCNPs was enhanced, which was beneficial for the further excitation of PS molecules and the improvement of the photodynamic efficacy. The thin silica layer would assist the sensitization function of ICG and the activation of triple PS molecules (Yue et al., 2018; Song et al., 2019). Thus, ICG as PS molecules and NIR sensitizer could effectively enhance PDT efficacy. The short half-life (<40 ns) and restricted action distance (<20 nm) of the generated ROSs usually limit the therapeutic efficacy of PDT (Thomas et al., 2017; Purushothaman et al., 2019). In this study, under the drive of mitochondria-targeting ligands, the obtained nanotheranostic agent would selectively accumulate in the mitochondria where ROSs were produced under the excitation of NIR laser and then the mitochondria-mediated cell apoptosis was induced (Scheme 1). Due to the enhanced luminescence intensity and high tissue-penetration depth of NIR laser, the developed multicolor upconversion nanotheranostic agent could achieve a significant inhibition effect on the deep-seated tumor.

## Materials and Methods

### Chemicals and Materials

Rare earth oxides, including Y_2_O_3_, Yb_2_O_3_, Tm_2_O_3_, and Ho_2_O_3_, were provided by the Sigma-Aldrich Corp while 1-octadecene (ODE), oleic acid (OA), and PS molecules were purchased from the Aladdin Reagent, Ltd. (Shanghai, China). The Sinopharm Chemical Reagent Co., Ltd. (Shanghai, China) offered other chemical reagents of analytical grade. An OKP purification system (Shanghai Laikie Instrument Corp., China) was used to prepare the aqueous solution. Mice were bought from Hubei Biossci Biotechnology Co., Ltd. (Wuhan, China) and the animal experiment was guided by the Animal Care and Use Committee of Linyi University.

### Characterizations

The size and morphology of the prepared nanoparticles were observed under the transmission electron microscope (TEM, model: JEM-2010, JEOL) and their crystalline phase was determined by the X-ray powder diffractometer (D8 ADVANCE, CuKα radiation, λ = 1.5405Å). A fluorescence spectrophotometer (mode: F-4600, Hitachi) equipped with an external NIR laser was used to obtain the upconversion luminescence spectra. The UV-Vis absorption spectra of the prepared nanoparticles were characterized with an Agilent UV-Vis spectrophotometer (model: Cary 60) and their ζ potentials were acquired with the Zeta-size nano instrument (Zen 3600, Malvern Instruments Ltd.). The two-photon laser scanning confocal microscope (model: Leica TCS SP5) was used to trace the fluorescence information and get the fluorescence images. The *in vitro* cytotoxicity of the obtained nanotheranostics was evaluated by the CCK-8 test which was performed on a Microplate Reader (Thermo Scientific Multi-skan Mk3). Flow cytometry (cytoflex, beckmancoulter, America) was used to analyze the cellular fluorescence information and cell apoptosis.

### Preparation of the Nanotheranostic Agent

#### Preparation of UCNPs

Based on the previously reported method, 0.05 mmol/L Y(oleate)_3_ and Ln(oleate)_3_ were prepared in OA/ODE mixing solution (v/v = 1:1) (Wei et al., 2006; Li et al., 2015). The molar ratio of lanthanide ions was Y:Yb:Tm:Ho = 54.5:40:0.5:5. Then, UCNPs with a NaYF_4_@NaYF_4_:Yb,Tm,Ho@NaYF_4_ structure were prepared via the layer-by-layer seed-mediated shell growth strategy (Song et al., 2017, 2018). Firstly, to prepare the NaYF_4_ core, 20.0 mL Y(oleate)_3_ solution and 0.84 g NaF were mixed and reacted at 110°C under the protection of argon (Ar) for 1.0 h, and then further reacted at 340°C for another 2.0 h. Secondly, 8.0 mL Ln(oleate)_3_ solution was slowly injected into the above solution and reacted at 340°C for 20 min to grow the luminescent shell on the surface of the NaYF_4_ core. Lastly, 12.0 mL Y(oleate)_3_ solution was added and reacted for another 20 min to prepare the outer shell NaYF_4_. The prepared UCNPs were obtained by precipitation in 2-fold volume of ethanol, centrifugally collected, and washed with hexane/ethanol (v/v = 1:6) for several times. The obtained UCNPs were finally dispersed in cyclohexane.

#### Preparation of UCNPs@SiO_2_/HA/MB/ICG

The silica layer grew on the surface of UCNPs via the water-in-oil reverse microemulsion method (Liu et al., 2014). The detailed preparation process was as follows: reverse micelles were first formed by homogeneously mixing Igepal CO-520 (0.660 mL) into cyclohexane (10.0 mL). Afterward, 0.450 mmol of the prepared oleic acid protected UCNPs was added into the above reverse micelles and strongly stirred for 1.0 h. Via the ligand exchange between oleate ligand and Igepal CO-520, UCNPs were entrapped in the water pool. Photosensitizer molecules including HA solution (90 μL, 5.0 mg/mL, ethanol), equal amount of ICG solution and MB aqueous solution were added in sequence. Then, 60 μL ammonia (30%) was added dropwise and stirred for 2.0 h to make the solution alkaline. Finally, 90 μL tetraethyl orthosilicate (TEOS) was slowly added into the solution and reacted for 24 h. Thus, the silica layer grew on the surface of UCNPs via the classic hydrolysis and condensation. Based on the proposed procedure, single PS molecules loaded nanoprobes and the control nanoprobe, UCNPs@SiO_2_, were prepared.

#### Preparation of UCNPs@SiO_2_/HA/MB/ICG@PEG-TPP

To enhance dispersibility, the obtained UCNPs@SiO_2_/HA/MB/ICG nanoprobe was modified with amino-PEG. 10.0 mg of the prepared UCNPs@SiO_2_/HA/MB/ICG nanoprobe was added into 5.0 mL of silance-PEG-NH_2_ and shook slowly for 12 h to obtain the UCNPs@SiO_2_/HA/MB/ICG@PEG. Then, the mitochondria-targeted ligand, 3-carboxypropyl triphenyl-phosphonium bromide (CTPB), was attached onto the surface of the prepared nanoprobe via the covalent reaction. 8.0 mg of the UCNPs@SiO_2_/HA/MB/ICG@PEG nanoprobe was collected by centrifugation, then redispersed in 8.0 mL of CTPB solution (0.86 mg/mL, methanol) and reacted for 12 h via the catalysis of 1-(3-dimethylaminopropyl)-3-ethylcarbodiimide hydrochloride (EDC, 3.46 mg/mL). The obtained UCNPs@SiO_2_/HA/MB/ICG@PEG-TPP nanotheranostic agent was washed with water several times and then stored in PBS buffer solution for further use.

### Evaluation of the Prepared Nanotheranostic Agent

#### Detection of the Produced ROSs in Aqueous Solution

The commonly used fluorescence dye, 9,10-anthracenediyl-bis(methylene)dimalonic acid (ABDA), could irreversibly react with ROSs to induce the decrease in its fluorescence intensity at 407 nm (Chen et al., 2016; Dong et al., 2016). Thus, the change of the fluorescence intensity of ABDA could be used to evaluate the generated ROSs in aqueous solution. ABDA (10 μM, DMSO) was added into 1.0 mg/mL of the prepared UCNPs@SiO_2_/HA/MB/ICG@PEG-TPP solution and the mixture was irradiated with NIR laser (980 nm, 1.5 W/cm^2^) for 21 min. Every irradiation lasted for 3 min with an interval of 1 min. After irradiation, ABDA was excited at 380 nm and its fluorescence was recorded at 407 nm. Each time point was operated five times (*n* = 5).

#### Cellular Uptake and Localization

To evaluate the cellular uptake and localization, MCF-7 cancer cells cultured in glass coverslips were incubated with 70 μg/mL of the UCNPs@SiO_2_/HA/MB/ICG@PEG-TPP nanotheranostic agent for different times. MCF-7 cancer cells were washed with PBS buffer several times and then imaged immediately with a two-photon laser confocal scanning microscope. The fluorescence images of the prepared UCNPs@SiO_2_/HA/MB/ICG@PEG-TPP nanotheranostic agent were recorded at the wavelength range of 515–575 nm and excited by the 980 nm laser while the mitochondria of MCF-7 cancer cells were stained with a commercial fluorescent dye, the MitoTracker®Deep Red. Fluorescence signal was recorded at the wavelength range of 650–720 nm under the excitation of 633 nm.

#### *In vitro* ROSs Detection

The *in vitro* ROSs was detected with the flow cytometry and laser confocal scanning microscope. For the flow cytometry, the prepared UCNPs@SiO_2_/HA/MB/ICG@PEG-TPP nanotheranostic agent (70 μg/mL) was used to incubate MCF-7 cancer cells for 12.0 h. Afterwards, MCF-7 cancer cells were washed with PBS buffer and then divided into two parallel subgroups, the experimental group, which was treated with laser, and the control group without the excitation. After being excited under the NIR laser (980 nm, 1.5 W/cm^2^) for 4 min, MCF-7 cancer cells were cultured for another 24 h. Then, the two parallel subgroups were collected and resuspended in 2′,7′-dichlorofluorescin diacetate staining solution (DCFH-DA, diluted with serum-free DMEM by 5,000-fold) for 30 min, washed with PBS and injected for flow cytometry. For *in-situ* observation of ROSs, MCF-7 cancer cells were stained with DCFH-DA solution (10 μM, serum-free DMEM) in a cell incubator for 30 min after incubation with the prepared nanotheranostic agent, and then excited under the laser. Then, MCF-7 cancer cells were washed with PBS buffer several times to remove excess dyes and then observed in 1.0 mL of PBS buffer under the scanning confocal microscope. Under an excitation of 488 nm, the green channel (500–540 nm) was used to obtain the fluorescence information of DCF which was the oxidization product of DCFH-DA by ROSs.

#### Observation of the Changes of the Mitochondrial Membrane Potential (ΔΨ_m_)

The ΔΨ_m_ changes were also detected with flow cytometry and laser confocal scanning microscope. MCF-7 cancer cells were treated with the procedure described for ROSs detection. After irradiation and incubation for further 24 h, the collected MCF-7 cancer cells were resuspended in 1.0 mL of serum-free DMEM containing 25 μL JC-1 staining solution for 20 min, washed with cold JC-1 buffer solution and used for flow cytometry. For *in-situ* observation of the ΔΨ_m_ changes, MCF-7 cancer cells were incubated with 1.0 mL of JC-1 staining solution in the cell incubator for 20 min, washed with cold JC-1 buffer solution and then imaged under the laser scanning confocal microscope. The fluorescence information of JC-1 was obtained at green channel (λ_ex_ = 488 nm, λ_em_ = 500–550 nm) and red channel (λ_ex_ = 561 nm, λ_em_ = 580–640 nm), respectively.

#### PDT Efficacy Assay in Living Cells

In this experiment, the *in vitro* PDT efficacy of the obtained nanotheranostic agent was examined with the CCK-8 assay and flow cytometry. Four parallel experiments were chosen. MCF-7 cancer cells were treated with (a) PBS, (b) the prepared nanotheranostic agent only, (c) laser irradiation (980 nm, 1.5 W/cm^2^, 4.0 min with an interval of 1.0 min), (d) the prepared nanotheranostic agent and laser irradiation. To process the CCK-8 assay, MCF-7 cancer cells cultured in the 96-well-microtiter plates were incubated with the obtained nanotheranostic agent (70 μg/mL). After 12 h, MCF-7 cancer cells were washed with PBS buffer, excited under the NIR laser and then incubated for another 24 h. Afterward, the CCK-8 agent (10 μL) was added into each pore and incubated cells for 2 h. Finally, the microplate reader was used to record the absorbance of MCF-7 cancer cells at 450 nm (Yue et al., 2018; Song et al., 2019).

The effects of the prepared nanotheranostic agent and NIR irradiation on the cytotoxicity were evaluated by the flow cytometry. MCF-7 cancer cells were treated with (a) PBS, (b) the prepared nanotheranostic agent (70 μg/mL, 12 h), (c) laser irradiation (980 nm, 1.5 W/cm^2^, 4.0 min with an interval of 1.0 min), (d) the prepared nanotheranostic agent and laser irradiation. After treatment, MCF-7 cancer cells were collected, stained with apoptosis staining solution for 5.0 min (500 μL binding buffer, 6 μL Annexin V-FITC staining solution and 7 μL PE staining solution) and then injected for flow cytometry analysis. Based on the manufacturer's instruction, the necessary fluorescence compensation was adjusted (Song et al., 2019).

#### *In vivo* PDT Efficacy Assay

The *in vivo* PDT efficacy of the prepared UCNPs@SiO_2_/HA/MB/ICG@PEG-TPP nanotheranostic agent was evaluated via animal experiment. The animal study was reviewed and approved by the Animal Care and Use Committee of Linyi University. First, cancer cells at the density of 1 × 10^6^ were injected into the Bald/c nude mouse (6 weeks, around 20 g). Experiments were performed when the tumors grew to the tumor volume of 100–130 mm^3^. To mimic the deep-seated tumor, a piece of mouse tissue with a thickness of 7 mm was covered on the tumor surface. The tumor-bearing mice were injected with the prepared nanotheranostic agent (0.6 mg/ml, 50 μL) and then their tumor section irradiated with the NIR laser after 12 h (980 nm, 1.5 W/cm^2^, 4.0 min with an interval of 1.0 min). Afterward, the tumor volume (V = length × width^2^/2) was recorded every 2 days. On the seventh day, the same amount of prepared nanotheranostic agent was injected into the mice again and then the tumor section was irradiated with the NIR laser. After 13 days, the mice were sacrificed to obtain the main organs for hematoxylin and eosin (H&E) staining and tumor section for H&E staining, TUNEL staining and Caspase-3 staining. To evaluate the PDT efficacy of the prepared nanotheranostic agent, three other parallel control groups were, respectively, (a) only injected with PBS, (b) laser irradiation alone, (c) only injected with the nanotheranostic agent.

## Results and Discussion

### Characterization of the UCNPs@SiO_2_/HA/MB/ICG@TPP Nanotheranostic Agent

**Figure 1:**
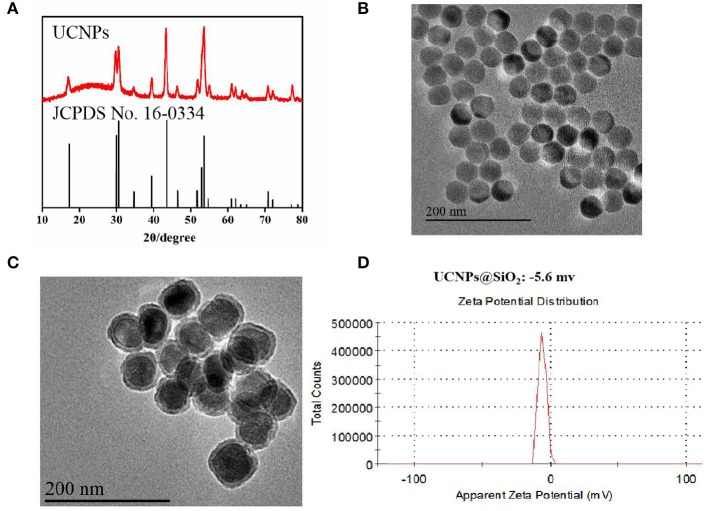
**(A)** XRD images of the prepared UCNPs; **(B)** TEM images of the prepared UCNPs; **(C)** TEM images of the prepared UCNPs@SiO_2_; **(D)** Zeta-potential of the prepared UCNPs@SiO_2_.

Since the sensitization effects of the ICG dye could alleviate the luminescence concentration quenching effect of Yb^3+^ (Wei et al., 2006), the doping ratio of the luminescence layer (Y:Yb:Tm:Ho = 54.5:40:0.5:5) was chosen to obtain multi-color UCNPs (Figure S1). Based on the layer-by-layer seed mediated shell growth strategy, UCNPs were obtained with pure hexagonal phase (Figure 1A) and homogeneous particles sizes (Figure 1B). Based on the modified water-in-oil reverse microemulsion method (Purushothaman et al., 2019), dense silica was homogeneously grown on the surface of UCNPs with a thickness of 6.2–8.3 nm (Figure 1C) with the finial zeta-potential of −5.6 mv (Figure 1D). During the silanization, PS molecules could be easily incorporated into the silica layer without further modification with a concentration of 19.6–26.8 μg/mg, which was confirmed by the UV-Vis analysis (Figure S2A) and zeta-potential (Figure S2B). In addition, the introduction of ICG could enhance the luminescence intensity of UCNPs, which could further improve the photodynamic efficacy (Figure S3).

**Figure 2:**
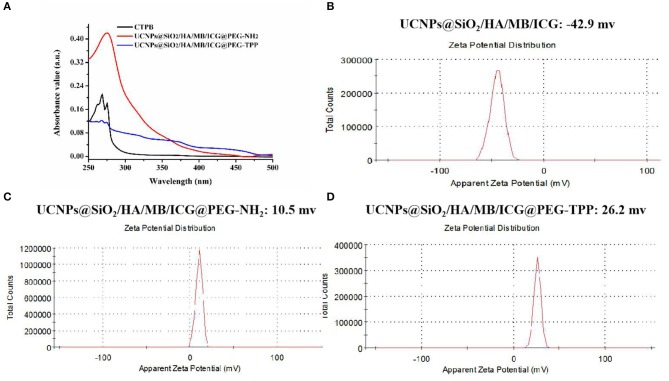
**(A)** Uv-Vis spectra of CTPB, UCNPs@SiO_2_/HA/MB/ICG@PEG-NH_2_, UCNPs@SiO_2_/HA/MB/ICG@PEG-TPP; Zeta-potential of **(B)** UCNPs@SiO_2_/HA/MB/ICG; **(C)** UCNPs@SiO_2_/HA/MB/ICG@PEG-NH_2_; **(D)** UCNPs@SiO_2_/HA/MB/ICG@PEG-TPP.

In this experiment, the prepared UCNPs@SiO_2_/HA/MB/ICG nanoprobe was coated with PEG-NH_2_ via the silanization reaction and then the mitochondria recognition ligand, TPP, was further attached through the carbodiimide reaction to obtain the final nanotheranostic agent, UCNPs@SiO_2_/HA/MB/ICG@PEG-TPP (Liu et al., 2014). As displayed in Figure 2A, the obtained UCNPs@SiO_2_/HA/MB/ICG@PEG-TPP showed the characteristic Uv-Vis peak of the TPP functional group, proving the satisfactory modification. The Zeta-potential of the UCNPs nanoprobe became positive when coated with PEG-NH_2_ (Figures 2B,C), and the mitochondria-targeting ligand, TPP, could further increase the Zeta-potential of the nanoprobe, which could benefit their accumulation in the mitochondria (Figure 2D).

### PDT Efficacy of the Prepared Nanotheranostic Agent in Aqueous Solution

**Figure 3:**
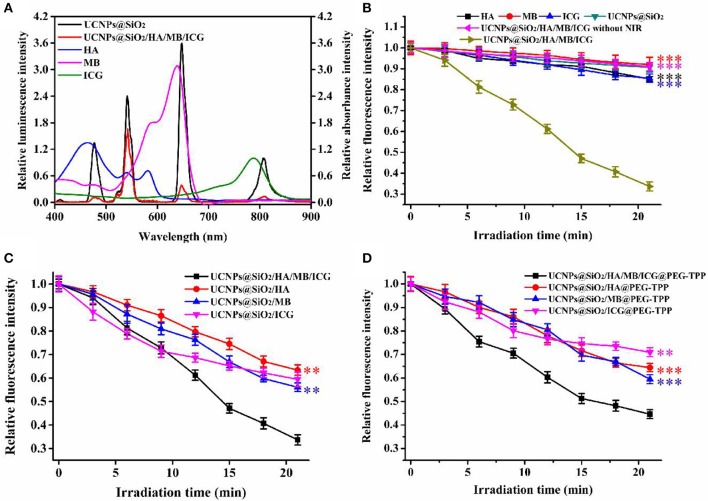
**(A)** Relative luminescence intensity of UCNPs@SiO_2_ and UCNPs@SiO_2_/HA/MB/ICG, relative absorbance value of HA, MB and ICG; **(B)** ROSs production of HA, MB, ICG, UCNPs@SiO_2_, UCNPs@SiO_2_/HA/MB/ICG under the irradiation of NIR laser (1.5 W/cm^2^), and UCNPs@SiO_2_/HA/MB/ICG without the irradiation; **(C)** ROSs production of the prepared nanoprobe under the irradiation of NIR laser; **(D)** ROSs production of the TPP modified nanoprobes under the irradiation of NIR laser. Each time point was operated five times and error bars represent standard deviation (*n* = 5). The data was analyzed by the *T*-test. **p < 0.01 and ***p < 0.001.

The shortened energy transfer distance and the well-matched spectra guaranteed a high energy transfer efficiency. As shown in Figure 3A and Figure S4, with the concentration of PS molecules increasing, the quenching yields increased to 91.4, 89.2, and 85.9% for luminescence peaking at 478, 648, and 808 nm, respectively. The ROSs indicator, 9,10-anthracenediyl-bis(methylene)dimalonic acid (ABDA), was further used to evaluate the ROSs generated in aqueous solution. As shown in Figure 3B, only the prepared nanotheranostic agent under the excitation of NIR laser could produce ROSs. By contrast, only PS molecules and the prepared UCNPs@SiO_2_ nanoparticles played a negligible effect on the fluorescence intensity of ABDA upon NIR irradiation. Similarly, the finial nanotheranostic agent in the absence of NIR irradiation did not have an obvious effect on the fluorescence intensity of ABDA. Thus, the final nanotheranostic agent and NIR were two indispensable parameters for the ROSs generation. Due to the cooperative photodynamic effects, ROSs produced by the prepared nanotheranostic agent could quench 66.3% of the fluorescence intensity of ABDA after 21.0 min of irradiation, which was much more than the corresponding single PS-involved nanoprobes and other reported UCNPs-based PDT nanoprobes (Figure 3C; Gnanasammandhan et al., 2016; He et al., 2016). After modification with TPP, the constructed nanotheranostic agent, UCNPs@SiO_2_/HA/MB/ICG@PEG-TPP, also showed obvious advantages in the production of ROSs (Figure 3D).

### Cellular Uptake and Localization

**Figure 4:**
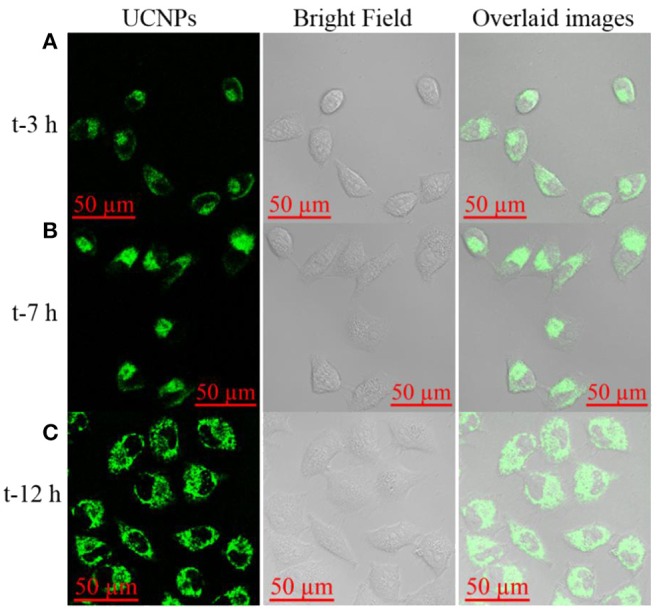
Upconversion luminescence imaging of MCF-7 cells treated with 70 μg/mL of the prepared nanoprobe for **(A)** 3 h, **(B)** 7 h, and **(C)** 12 h. Green channel was collected the 515–575 nm under the excitation of NIR laser to obtain the UCNPs information; Bright field was used to obtain the cell information.

To validate the application of the prepared nanotheranostic agent in the cell imaging and PDT, its cytotoxicity was first studied with the CCK-8 assay. As shown in Figure S5, MCF-7 cancer cells could keep above 95% of cell viability when treated with 0–0.125 mg/mL of the prepared nanotheranostic agent. The cell biocompatibility and distribution were observed under the two-photon confocal lasers scanning microscope. As demonstrated in Figure 4, the prepared nanotheranostic agent was firstly endocytosed into MCF-7 cancer cells and gradually captured in the lysosome/endosome in the first 7 h. With the incubation time prolonged to 12 h, the nanotheranostic agent was successfully escaped and released into the cytoplasm.

**Figure 5:**
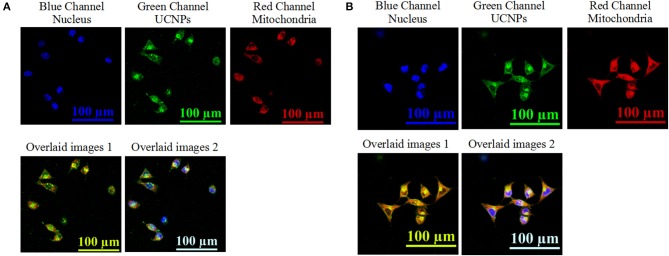
Upconversion luminescence imaging of MCF-7 cells treated with 70 μg/mL of **(A)** the prepared UCNPs@SiO_2_/HA/MB/ICG, **(B)** UCNPs@SiO_2_/HA/MB/ICG@PEG-TPP. The nucleus information was collected at the blue channel from 420 nm to 480 nm under the excitation of 405 nm laser; the UCNPs information was collected at the green channel from 515 to 575 nm under the excitation of NIR laser; the mitochondria information was collected at the red channel form 650 to 720 nm under the excitation of 633 nm; overlaid image 1 consisted of the green channel and red channel; overlaid image 2 consisted of the blue channel, green channel, and red channel.

The localization of the prepared nanotheranostic agent within cells was further evaluated. As illustrated in Figure 5, the prepared UCNPs@SiO_2_/HA/MB/ICG nanoprobe was mainly distributed in the cytoplasm (Figure 5A). When compared to the UCNPs@SiO_2_/HA/MB/ICG@PEG-TPP nanoprobe treated groups (Figure 5B), the fluorescence signals of the UCNPs and MitoTracker dye were overlapped well, proving that the functional group, TPP, would drive the prepared UCNPs@SiO_2_/HA/MB/ICG@PEG-TPP nanotheranostic agent to accumulate in the mitochondria where the *in-situ* PDT was achieved.

### Intracellular ROSs Generation

**Figure 6:**
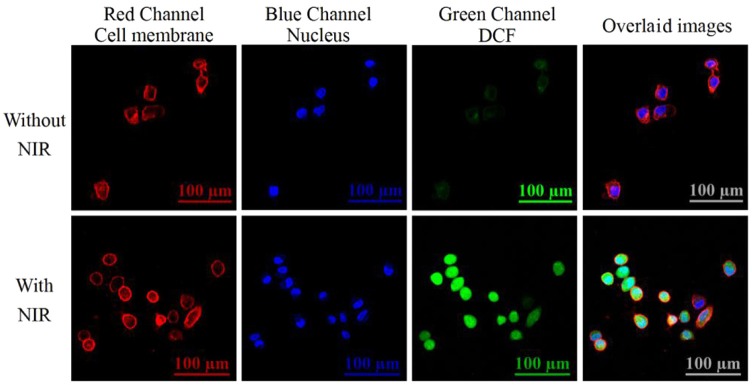
Confocal lasers scanning microscope (CLSM) images of intracellular ROSs. MCF-7 cancer cells were incubated with 70 μg/mL of the prepared nanotheranostic agent for 12 h. The cell membrane information was collected at the red channel from 600 to 650 nm under the excitation of 561 nm laser. The nucleus information was collected at the blue channel from 420 to 480 nm under the excitation of 405 nm laser; The ROSs information was collected at the green channel from 500 to 540 nm under the excitation of 488 nm. Overlaid images consisted of the three channels.

We further investigated the ability of the internalized nanotheranostic agent to produce ROSs in living cells with the fluorescent dye, 2,7-dichlorofluorescin diacetate (DCFH-DA). Once diffused into the cells, DCFH-DA as a cell-permeable oxidant-sensing probe would be converted into DCFH by related esterase, and then oxidized to DCF by ROSs which would emit bright green fluorescence when excited (Kim et al., 2014; Hou et al., 2016). Thus, the generation of ROSs would be reflected by the fluorescence signal of DCF which was recorded and imaged with the flow cytometry and confocal microscope (Figure 6 and Figure S6). As shown in Figure S6, MCF-7 cancer cells did not display obvious increased green fluorescence when only irradiated with the NIR laser or only incubated with the nanoprobe, proving the applicability of the used NIR laser (irradiation intensity: 1.5 W/cm^2^, irradiation time: 4 min with an interval of 1.0 min) for PDT and negligible cytotoxicity of the prepared nanotheranostic agent. By contrast, the internalized nanotheranostic agent could produce intracellular ROSs under the irradiation of NIR laser. As shown in the confocal laser scanning microscope images (Figure 6), the irradiation of NIR laser could activate the UCNPs@SiO_2_/HA/MB/ICG@PEG-TPP nanoprobe distributed in the mitochondria of MCF-7 cancer cells to produce ROSs which could oxidize DCFH into DCF with bright green fluorescence when excited.

### Mitochondrial Membrane Potential Measurement

**Figure 7:**
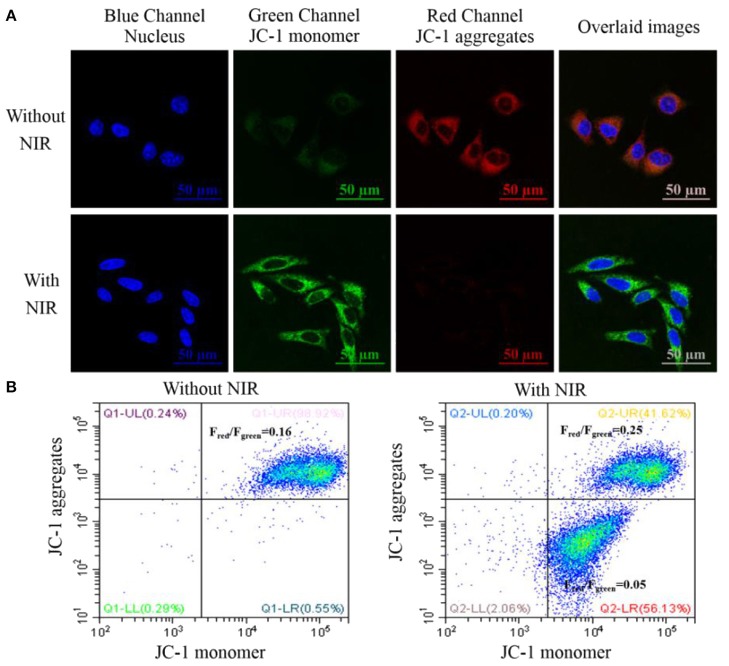
**(A)** CLSM images of intracellular mitochondrial membrane potential stained with the JC-1 dye. MCF-7 cancer cells were incubated with 70 μg/mL of the prepared nanotheranostic agent for 12 h. The nucleus information was recorded at the blue channel from 420 to 480 nm under the excitation of 405 nm. The fluorescence signal of the monomeric JC-1 dye was recorded at the green channel from 500 to 550 nm under the excitation of 488 nm; the fluorescence signal of the aggregated JC-1 dye was recorded at the red channel from 580 to 640 nm under the excitation of 561 nm; overlaid images of the three channels; **(B)** flow cytometry to evaluate the ΔΨ_m_ change of MCF-7 cancer cells using JC-1 staining.

PDT was shown to induce mitochondrial-dependent cell apoptosis which was related to the release of the pro-apoptotic proteins and caspase activation. The mitochondrial membrane potential (ΔΨ_m_) plays an important role in the biological activities in mitochondria and its decrease is an important indicator to assess the dysfunction of mitochondria (Crompton, 1999; Liu et al., 2014; Liu Y. Y. et al., 2016). In this experiment, the ΔΨ_m_ change could be studied with JC-1 fluorescence dye which would tend to aggregate in red fluorescence with high ΔΨ_m_ and become monomeric in green fluorescence with low ΔΨ_m_. Thus, the change of its fluorescence intensity (F_red_/F_green_) would reflect the mitochondrial membrane status. As shown in Figure 7A, JC-1 dye in the group only treated with the nanotheranostic agent would display weak green and strong red fluorescence while JC-1 dye in the group treated with the nanotheranostic agent and NIR laser would display stronger green and weaker red fluorescence, which was attributed to the decreased ΔΨ_m_ by the produced ROSs. The decrease in ΔΨ_m_ was further evaluated with flow cytometry (Figure 7B). In absence of NIR laser irradiation, F_red_/F_green_ was around 0.16 while about 56.13% of MCF-7 cells were moved into the below quadrant with a large decrease in F_red_/F_green_ ratio (0.05) after excited with NIR laser.

### Cancer Cell Apoptosis Induced by the Prepared Nanoprobe

**Figure 8:**
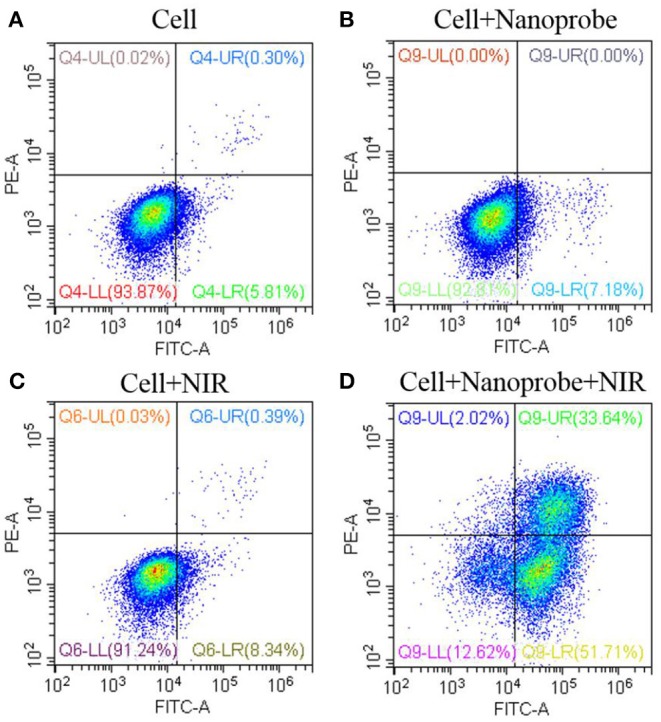
Cell viability (%) of the UCNPs@SiO_2_/HA/MB/ICG@PEG-TPP nanotheranostic agent treated with **(A)** PBS, **(B)** nanoprobe, **(C)** NIR irradiation, **(D)** nanoprobe and NIR irradiation.

Based on the generated ROSs and ΔΨ_m_ impair, mitochondrial-dependent cell apoptosis was expected. The cell apoptosis was first evaluated with the CCK-8 assay. As demonstrated in Figure S7, the NIR irradiation or the nanoprobe alone would have no significant influence on the cell viability. However, when treated with the designed photodynamic nanotheranostic agent, MCF-7 cancer cells could only retain 17.3% of cell viability. Moreover, the introduced triple PS molecules and designed photosensitizers-modulated sensitizing switch would improve the generation of ROSs and bring higher PDT efficacy. Moreover, the cell apoptosis was further analyzed by the flow cytometry which could distinguish the cells with high viability, early apoptosis, late apoptosis, or necrosis. In the control groups, more than 91% of cancer cells kept high viability and were located in the lower left quadrant (Figure 8). After being incubated with the prepared nanotheranostic agent and excited by the NIR laser, around 51.71 and 33.64% of cancer cells shifted from the high viability to early apoptosis and late apoptosis, respectively. Thus, the apoptosis was a major cell death modality induced by the prepared nanotheranostic agent.

### *In vivo* PDT Efficacy of the Prepared Nanotheranostic Agent

**Figure 9:**
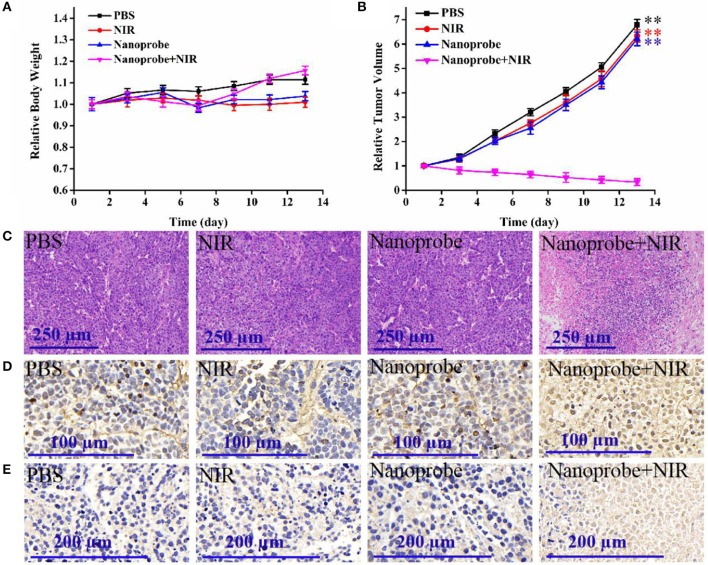
**(A)** Time-dependent mice body weight curves and **(B)** tumor growth curves of different groups of mice with various treatments, **(C)** H&E staining, **(D)** TUNEL staining, and **(E)** caspase-3 staining of the dissected tumor section on the thirteenth day with different treatments. Four parallel experiments were operated, and error bars represent standard deviation (*n* = 4). The data was analyzed by the *T*-test. **p < 0.01.

Furthermore, the *in vivo* therapeutic effect of the developed nanotheranostic agent was studied on xenograft mice. The experiment began when the tumor-bearing nude mice displayed tumor volumes of 100–130 mm^3^. Four groups with different treatments were designed: (a) injected with PBS buffer solution, (b) injected with the prepared nanotheranostic agent, (c) irradiated with NIR laser, (d) injected with the prepared nanotheranostic agent and then irradiated with the NIR laser. Every 2 days, we measured and recorded the changes in animal weight and tumor volume. There were no significant weight loss (Figure 9A) nor obvious tissue abnormalities recorded in the H&E staining in all groups (Figure S8), proving the minimal systemic toxicity of the prepared nanotheranostic agent. As expected, tumors in group b and group c increased by 6.2- to 6.3-fold with a similar growth rate to those in group a (Figure 9B and Figure S9). Due to the *in-situ* synergistic PDT efficacy, the prepared nanotheranostic agent would remarkably inhibit the tumor growth without recurrence which would finally induce 66% decrease of the tumor volume. The PDT efficacy of the prepared nanotheranostic agent was further verified by the histological results. When compared, tumor sections in group 4 displayed obvious blank areas as well as nuclear shrinkage and fragmentation due to the PDT efficacy (Figure 9C). Furthermore, the cell death mechanism induced by the prepared nanotheranostic agent was further studied with the TUNEL staining and Caspase-3 staining. As shown in Figure 9D, most cancer cells in the tumor section of groups a-c kept their spherical nuclei intact, thus the corresponding treatment did not affect the normal growth of the tumor. Contrarily, an increased amount of cancer cells displayed TUNEL positive nuclei in group d due to DNA fragmentation. The tumor section was further analyzed with the Caspase-3 staining. Consistent with TUNEL staining, cancer cells in the tumor section of group d also displayed positive Caspase-3 staining, implying that the produced ROSs could induce Caspase-mode cell apoptosis which involved the release of cytochrome c from the mitochondria to the cytosol, caspase activation, and other relative events leading to apoptosis (Figure 9E).

## Conclusions

Herein, we have developed a multi-color UCNPs-based nanotheranostic agent. Attributed to the ICG sensitization, multi-color UCNPs with high doping ratio of activators were prepared and used for loading triple PS molecules. The controlled thin silica layer could shorten the energy transfer distance, assist the sensitization function of ICG and activation of photosensitizer molecules. Thus, ROSs generated by the prepared nanotheranostic agent could quench 66.3% of the fluorescence intensity of ABDA after 21.0 min of irradiation. The modification of mitochondria-targeting ligand, TPP, drove the prepared nanotheranostic agent to accumulate in the mitochondria where ROSs were generated *in-situ*, and a high mitochondria-mediated cancer cells apoptosis (cell apoptosis ratio, 85.3%) was induced. Since the designed photodynamic nanotheranostic agent could produce increased intracellular ROSs, make the mitochondria dysfunction and induce cell apoptosis, it showed an obvious suppression effect on the deep-seated malignant tumors which would finally induce a 66% decrease of the tumor volume without obvious normal tissue impair and biotoxicity. Therefore, the developed nanotheranostic agent would act as an effective UCNPs-PDT nanoplatform and has great potential for the treatment of the deep-seated malignant tumors.

## Data Availability Statement

The raw data supporting the conclusions of this article will be made available by the authors, without undue reservation, to any qualified researcher.

## Ethics Statement

This animal study was reviewed and approved by the Animal Care and Use Committee of Linyi University.

## Author Contributions

TH and YJ operated this work. ZY performed the statistical analysis. XS and ZW contributed to the design of the study and wrote this manuscript. SZ contributed to the conception of this study.

### Conflict of Interest

The authors declare that the research was conducted in the absence of any commercial or financial relationships that could be construed as a potential conflict of interest.

## References

[B1] Chatterjee D. K. Fong L. S. Zhang Y. (2018). Nanoparticles in photodynamic therapy: an emerging paradigm. Adv. Drug Deliv. Rev. 60, 1627–1637. 10.1016/j.addr.2008.08.003 18930086

[B2] Chen C. W. Chan Y. C. Hsiao M. Liu R. S. (2016). Plasmon-enhanced photodynamic cancer therapy by upconversion nanoparticles conjugated with Au nanorods. ACS Appl. Mater. Inter. 8, 32108–32119. 10.1021/acsami.6b07770 27933825

[B3] Chen D. X. Tao R. Tao K. Chen B. Q. Choi S. K. Tian Q.. (2017). Efficacy dependence of photodynamic therapy mediated by upconversion nanoparticles: subcellular positioning and irradiation productivity. Small 13:1602053. 10.1002/smll.201602053 28060457

[B4] Chen G. Damasco J. Qiu H. Shao W. Ohulchanskyy T. Y. Valiev R. R.. (2015). Energy-cascaded upconversion in an organic dye-sensitized core/shell fluoride nanocrystal. Nano Lett. 15, 7400–7407. 10.1021/acs.nanolett.5b02830 26487489 PMC4915588

[B5] Crompton M. (1999). The mitochondrial permeability transition pore and its role in cell death. Biochem. J. 341, 233–249. 10.1042/0264-6021:3410233 10393078 PMC1220352

[B6] Dong C. Liu Z. Wang S. Zheng B. Guo W. Yang W.. (2016). A protein-polymer bioconjugate-coated upconversion nanosystem for simultaneous tumor cell imaging, photodynamic therapy, and chemotherapy. ACS Appl. Mater. Inter. 8, 32688–32698. 10.1021/acsami.6b11803 27934134

[B7] Fan W. Shen B. Bu W. Chen F. He Q. Zhao K.. (2014). A smart upconversion-based mesoporous silica nanotheranostic system for synergetic chemo-/radio-/photodynamic therapy and simultaneous MR/UCL imaging. Biomaterials 35, 8992–9002. 10.1016/j.biomaterials.2014.07.024 25103233

[B8] Garfield D. J. Borys N. J. Hamed S. M. Torquato N. A. Tajon C. A. Tian B. (2018). Enrichment of molecular antenna triplets amplifies upconverting nanoparticle emission. Nat. Photonics 12, 402–407. 10.1038/s41566-018-0156-x

[B9] Ge X. Q. Liu J. Sun L. (2017). Controlled optical characteristics of lanthanide doped upconversion nanoparticles for emerging applications. Dalton Trans. 46, 16729–16737. 10.1039/c7dt03049e 29125162

[B10] Gnanasammandhan M. K. Idris N. M. Bansal A. Huang K. Zhang Y. (2016). Near-IR photoactivation using mesoporous silica-coated NaYF_4_:Yb,Er/Tm upconversion nanoparticles. Nat. Protoc. 11, 688–713. 10.1038/nprot.2016.035 26963631

[B11] He L. C. Dragavon J. Cho S. Y. Mao C. C. Yildirim A. Ma K. (2016). Self-assembled gold nanostar-NaYF_4_:Yb/Er clusters for multimodal imaging, photothermal and photodynamic therapy. J. Mater. Chem. B 4, 4455–4461. 10.1039/c6tb00914j 32263428

[B12] Hou Z. Y. Deng K. R. Li C. X. Deng X. R. Lian H. Z. Cheng Z. Y. (2016). 808 nm Light-triggered and hyaluronic acid-targeted dual-photosensitizers nanoplatform by fully utilizing Nd^3+^-sensitized upconversion emission with enhanced anti-tumor efficacy. Biomaterials 101, 32–46. 10.1016/j.biomaterials.2016.05.024 27267626

[B13] Huang P. Rong P. Jin A. Yan X. Zhang M. G. Lin J.. (2014). Dye-loaded ferritin nanocages for multimodal imaging and photothermal therapy. Adv. Mater. 26, 6401–6408. 10.1002/adma.201400914 25123089 PMC4215197

[B14] Kim J. Santos Q. A. Park J. H. (2014). Selective photosensitizer delivery into plasma membrane for effective photodynamic therapy. J. Control. Release 191, 98–104. 10.1016/j.jconrel.2014.05.049 24892975

[B15] Lee J. Yoo B. Lee H. Cha G. D. Lee H. S. Cho Y.. (2017). Ultra-wideband multi-dye-sensitized upconverting nanoparticles for information security application. Adv. Mater. 29:1603169. 10.1002/adma.201603169 27748544

[B16] Li Z. Lv S. Wang Y. Chen Y. Liu Z. (2015). Construction of LRET-based nanoprobe using upconversion nanoparticles with confined emitters and bared surface as luminophore. J. Am. Chem. Soc. 137, 3421–3427. 10.1021/jacs.5b01504 25707940

[B17] Liang T. Li Z. Wang P. Zhao F. Liu J. Liu Z. (2018). Breaking through the signal-to-background limit of upconversion nanoprobes using a target-modulated sensitizing switch. J. Am. Chem. Soc. 140, 14696–14703. 10.1021/jacs.8b07329 30362727

[B18] Liu B. Li C. X. Yang D. M. Hou Z. Y. Ma P. A. Cheng Z. Y. (2014). Upconversion-luminescent core/mesoporous-silica-shell structured beta-NaYF_4_:Yb^3+^,Er^3+^@SiO_2_@mSiO_2_ composite nanospheres: fabrication and drug-storage/release properties. Eur. J. Inorg. Chem. 11, 1906–1913. 10.1002/ejic.201301460

[B19] Liu Y. Kang N. Lv J. Zhou Z. Zhao Q. Ma L.. (2016). Deep photoacoustic/luminescence/magnetic resonance multimodal imaging in living subjects using high-efficiency upconversion nanocomposites. Adv. Mater. 28, 6411–6419. 10.1002/adma.201506460 27185066

[B20] Liu Y. Liu Y. Bu W. Cheng C. Zuo C. Xiao Q.. (2015). Hypoxia induced by upconversion-based photodynamic therapy: towards highly effective synergistic bioreductive therapy in tumors. Angew. Chem. Int. Ed. 54, 8105–8109. 10.1002/anie.201500478 26012928

[B21] Liu Y. Y. Zhang J. W. Zuo C. J. Zhang Z. Ni D. L. Zhang C. (2016). Upconversion nano-photosensitizer targeting into mitochondria for cancer apoptosis induction and cyt c fluorescence monitoring. Nano Res. 9, 3257–3266. 10.1007/s12274-016-1204-9

[B22] Lv R. Wang D. Xiao L. Chen G. Xia J. Prasad P. N. (2017). Stable ICG-loaded upconversion nanoparticles: silica core/shell theranostic nanoplatform for dual-modal upconversion and photoacoustic imaging together with photothermal therapy. Sci. Rep. 7:15753. 10.1038/s41598-017-16016-x 29147000 PMC5691150

[B23] Purushothaman B. Choi J. Park S. Lee J. J. Samson A. A. S. Hong S. (2019). Biotin-conjugated PEGylated porphyrin self-assembled nanoparticles co-targeting mitochondria and lysosomes for advanced chemo-photodynamic combination therapy. J. Mater. Chem. B 7, 65–79. 10.1039/c8tb01923a 32254951

[B24] Song X. Yue Z. Hong T. Wang Z. Zhang S. (2019). Sandwich-structured upconversion nanoprobes coated with a thin silica layer for mitochondria-targeted cooperative photodynamic therapy for solid malignant tumors. Anal. Chem. 91, 8549–8557. 10.1021/acs.analchem.9b01805 31247732

[B25] Song X. Zhang J. Yue Z. Wang Z. Liu Z. Zhang S. (2017). Dual-activator codoped upconversion nanoprobe with core multishell structure for *in vitro* and *in vivo* detection of hydroxyl radical. Anal. Chem. 89, 11021–11026. 10.1021/acs.analchem.7b02995 28920422

[B26] Song X. Y. Yue Z. H. Zhang J. Y. Jiang Y. X. L. Wang Z. H. Zhang S. S. (2018). Multicolor upconversion nanoprobes based on a dual luminescence resonance energy transfer assay for simultaneous detection and bioimaging of [Ca^2+^]_i_ and pH_i_ in living cells. Chem. Eur. J. 24, 6458–6463. 10.1002/chem.201800154 29488255

[B27] Thomas A. P. Palanikumar L. Jeena M. T. Kim K. Ryu J. H. (2017). Cancer-mitochondria-targeted photodynamic therapy with supramolecular assembly of HA and a water soluble NIR cyanine dye. Chem. Sci. 8, 8351–8356. 10.1039/c7sc03169f 29619181 PMC5858757

[B28] Wang X. Valiev R. R. Ohulchanskyy T. Y. Ågren H. Yang C. Chen G. (2017). Dye-sensitized lanthanide-doped upconversion nanoparticles. Chem. Soc. Rev. 46, 4150–4167. 10.1039/c7cs00053g 28621356

[B29] Wei Y. Lu F. Q. Zhang X. R. Chen D. P. (2006). Synthesis of oil-dispersible hexagonal-phase and hexagonal-shaped NaYF_4_:Yb,Er nanoplates. Chem. Mater. 18, 5733–5737. 10.1021/cm0606171

[B30] Wu X. Zhang Y. Takle K. Bilsel O. Li Z. Lee H.. (2016). Dye-sensitized core/active shell upconversion nanoparticles for optogenetics and bioimaging applications. ACS Nano 10, 1060–1066. 10.1021/acsnano.5b06383 26736013 PMC4913696

[B31] Xu J. Yang P. Sun M. Bi H. Liu B. Yang D.. (2017). Highly emissive dye-sensitized upconversion nanostructure for dual-photosensitizer photodynamic therapy and bioimaging. ACS Nano 11, 4133–4144. 10.1021/acsnano.7b00944 28320205

[B32] Yan F. Wu H. Liu H. Deng Z. Liu H. Duan W.. (2016). Molecular imaging-guided photothermal/photodynamic therapy against tumor by iRGD-modified indocyanine green nanoparticles. J. Control. Release 224, 217–228. 10.1016/j.jconrel.2015.12.050 26739551

[B33] Yue Z. Hong T. Song X. Wang Z. (2018). Construction of a targeted photodynamic nanotheranostic agent using upconversion nanoparticles coated with an ultrathin silica layer. Chem. Commun. 54, 10618–10621. 10.1039/c8cc05121f 30177988

[B34] Zheng M. Yue C. Ma Y. Gong P. Zhao P. Zheng C.. (2013). Single-step assembly of DOX/ICG loaded lipid-polymer nanoparticles for highly effective chemo-photothermal combination therapy. ACS Nano 7, 2056–2067. 10.1021/nn400334y 23413798

[B35] Zou W. Visser C. Maduro J. A. Pshenichnikov M. S. Hummelen J. C. (2012). Broadband dye-sensitized upconversion of near-infrared light. Nat. Photonics 6, 560–564. 10.1038/nphoton.2012.158

[B36] Zou X. Xu M. Yuan W. Wang Q. Shi Y. Feng W.. (2016). A water-dispersible dye-sensitized upconversion nanocomposite modified with phosphatidylcholine for lymphatic imaging. Chem. Commun. 52, 13389–13392. 10.1039/c6cc07180e 27786316

