# Supplementary material for: Construction of Multicolor Upconversion Nanotheranostic Agent for in-situ Cooperative Photodynamic Therapy for Deep-Seated Malignant Tumors
Source: Front Chem. 2020 Feb 11;8:52. doi: 10.3389/fchem.2020.00052 (PMC7026389; doi:10.3389/fchem.2020.00052)
Supplement: Supplementary file 1 [file Data_Sheet_1.DOCX]

Supplementary Material

**Contents**

[1. Characterization of the prepared UCNPs 3](#_Toc13277)

[2. Characterization of the prepared UCNPs@SiO](#_Toc30111)_[2](#_Toc30111)_[/HA/MB/ICG nanoprobe 3](#_Toc30111)

[3. Characterization of the prepared UCNPs@SiO](#_Toc19091)_[2](#_Toc19091)_[/ICG nanoprobe 4](#_Toc19091)

[4. Optimization of the concentration of PS molecules 5](#_Toc7823)

[5. Cytotoxicity evaluation 5](#_Toc19970)

[6.](#_Toc16634) *[In vitro](#_Toc16634)* [ROSs detection 6](#_Toc16634)

[7. PDT efficacy assay in living cells 7](#_Toc19977)

[8. H&E staining analysis 8](#_Toc20370)

[9. Animal experiments 9](#_Toc14206)

[Reference 10](#_Toc18489)

1. **Characterization of the prepared UCNPs**

Since the sensitization effects of the ICG dye could alleviate the luminescence concentration quenching effect of Yb^3+^ (Wei et al., 2016), the doping ratio of the luminescence layer (Y:Yb:Tm:Ho=54.5:40:0.5:5) was chosen to obtain multi-color UCNPs

**
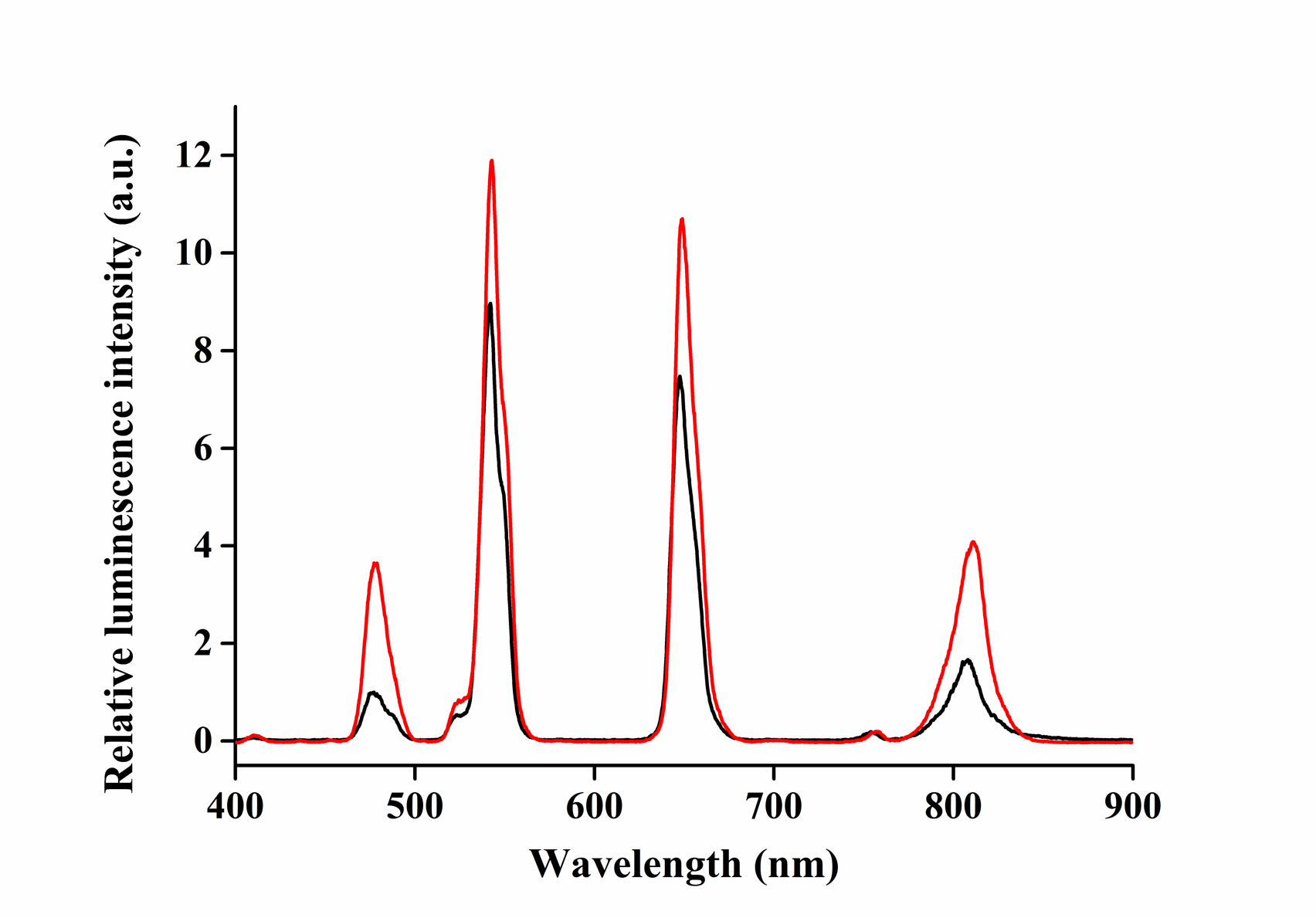
**

Figure S1: Relative luminescence intensity of UCNPs, the loaded molar ratios of the medium luminescence layer were Y:Yb:Tm:Ho=54.5:40:0.5:5 (red line) and Y:Yb:Tm:Ho=77.8:20:0.2:2 (black line), respectively.

1. **Characterization of the prepared UCNPs@SiO_2_/HA/MB/ICG nanoprobe**

Via the water-in-oil reverse microemulsion method, the silica layer grew on the surface of the prepared UCNPs (Liu et al., 2014). During the silanization, PS molecules could be easily incorporated into the silica layer without further modification. As shown in Figure S2a, the prepared UCNPs@SiO_2_/HA/MB/ICG nanoprobe displayed the characteristic Uv-Vis peaks of the three PS molecules. Calculated by the Uv-Vis analysis, the loading amount of HA, MB and ICG was 19.6±0.94 µg/mg, 26.8±1.29 µg/mg and 22.4±1.16 µg/mg, respectively. Moreover, the loading of the three PS molecules could endow the prepared nanoprobe with the increased negative potential value (Figure S2b).


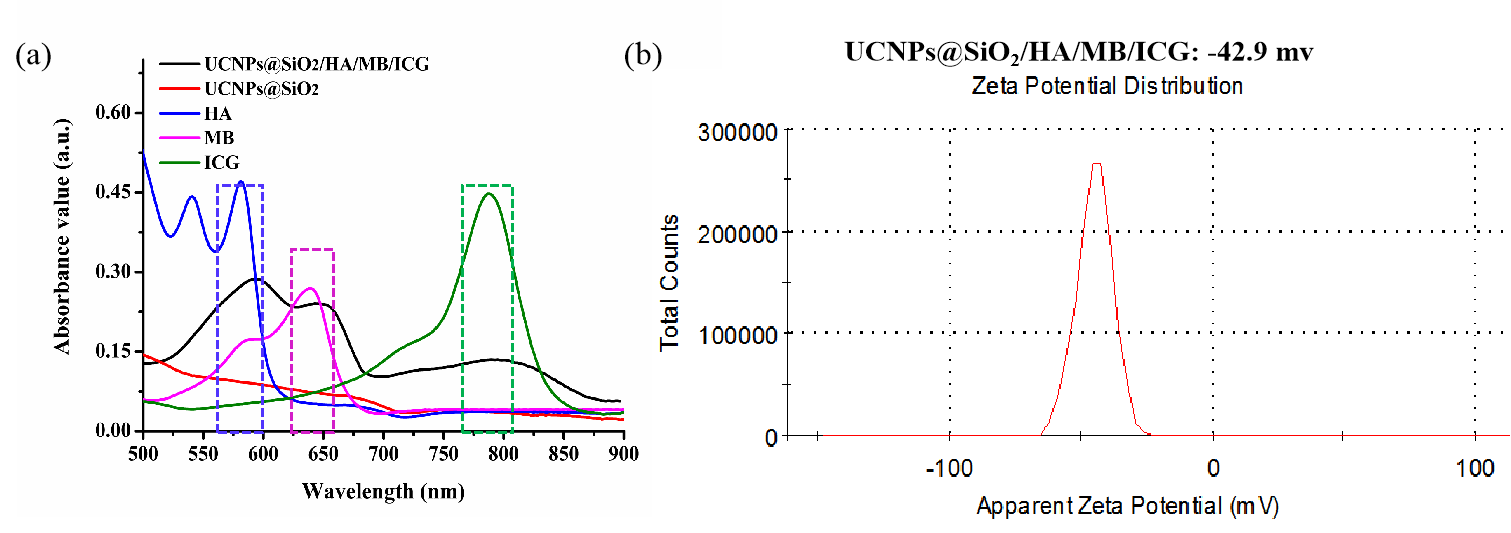


Figure S2: (a) Uv-Vis spectra of UCNPs@SiO_2_/HA/MB/ICG, UCNPs@SiO_2_, HA, MB, ICG; (b) Zeta-potential analysis of UCNPs@SiO_2_/HA/MB/ICG.

1. **Characterization of the prepared UCNPs@SiO_2_/ICG nanoprobe**

As shown in Figure S3, since ICG could effectively sensitize Yb^3+^ based on the Förster-type energy transfer, the introduction of indocyanine green (ICG) could enhance the luminescence intensity of the nanoprobe (Yan et al., 2016).


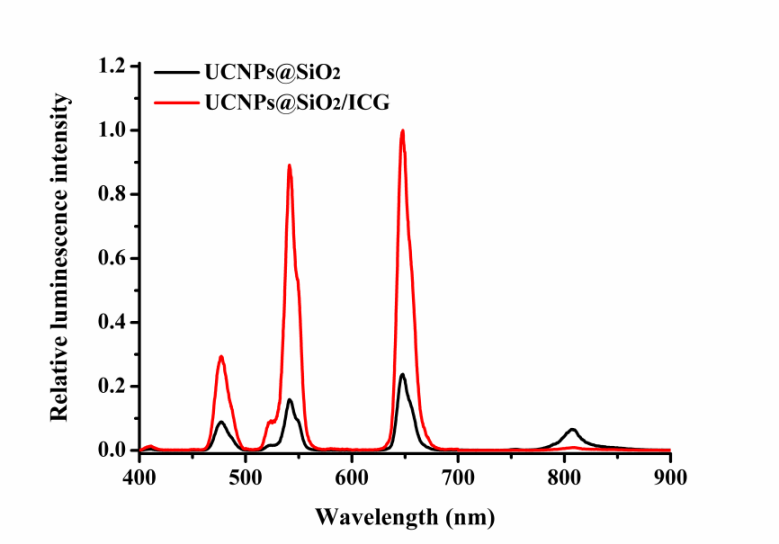


Figure S3: The relative luminescence spectra of UCNPs@SiO_2_ and UCNPs@SiO_2_/ICG.

1. **Optimization of the concentration of PS molecules**

With the concentration of PS molecules increasing, the quenching yields were increased to 91.4%, 89.2%, and 85.9% for luminescence peaking at 478 nm, 648 nm and 808 nm, respectively (Figure S4).


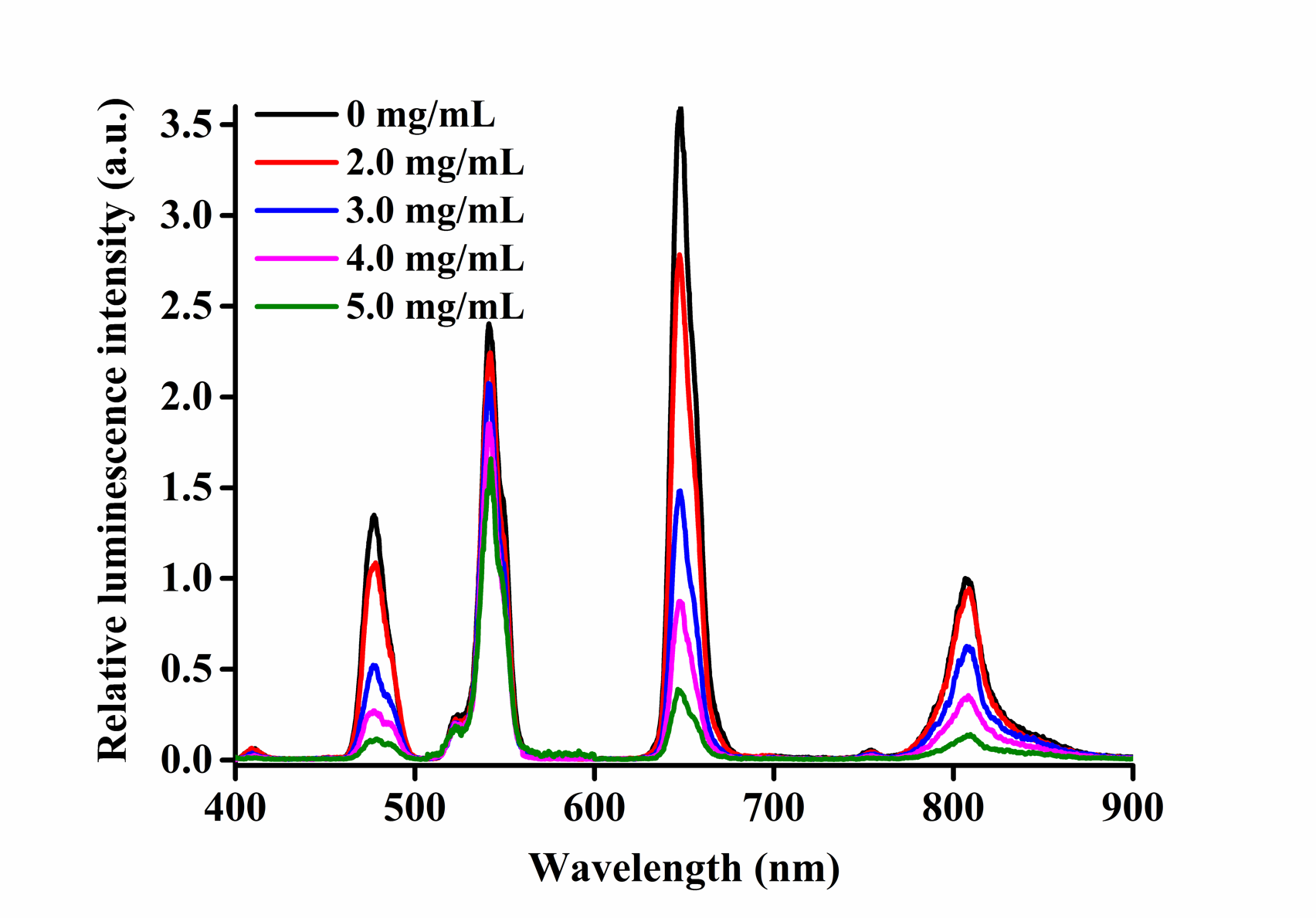


Figure S4: The relative luminescence spectra of UCNPs@SiO_2_/HA/MB/ICG prepared with different concentrations of PS molecules. The concentration of PS molecules was equal in each group.

1. **Cytotoxicity evaluation**

The cell culture solution contained 90% Dulbecco’s modified Eagle’s medium (DMEM), 10% heat-inactivated fetal bovine serum (FBS), penicillin (100 U/mL), and streptomycin (100 U/mL). MCF-7 cells were cultured in the above culture solution at 37^o^C in humidified air containing 5% CO_2_. For the CCK-8 assay, MCF-7 cancer cells (15000 cells per well) were transferred and then cultured in 96-well flat-bottom microtiter plates. Then, the prepared UCNPs@SiO_2_/HA/MB/ICG@PEG-TPP nanotheranostic agent in the concentration range of 0-0.15 mg/mL were added into the corresponding wells, respectively, and four parallel wells were used to examine each concentration. MCF-7 cancer cells was washed with PBS buffer solution three times after being incubated with the nanotheranostic agent for 24 h. Then, 10 µL of CCK-8 agent was added into each well and incubated MCF-7 cancer cells at 37^o^C for another 2 h. Finally, the absorbance of MCF-7 cancer cells at 450 nm were recorded with the microplate reader. Cell viability (%) was calculated according to the equation: Cell Viability (%) = (Mean Absorbance_treated wells_-Mean Absorbance_blank wells_)/ (Mean Absorbance_control wells_-Mean Absorbance_blank wells_)×100% (Song et al., 2019; Yue et al., 2018) . Each concentration was operated five times (n=5).


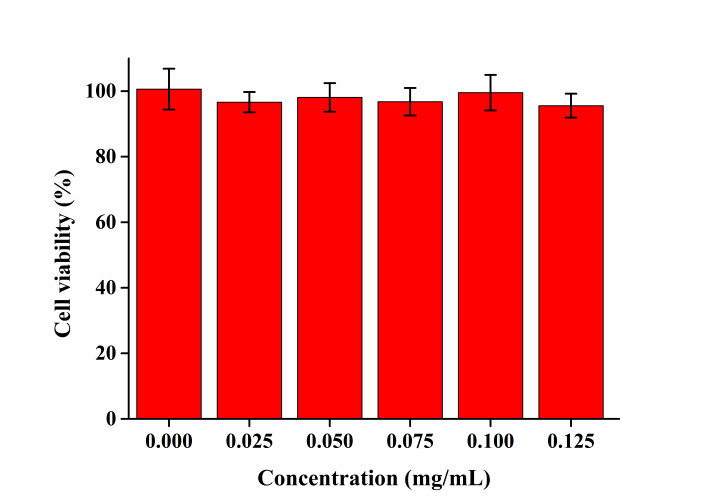
As demonstrated in Figure S5, MCF-7 cancer cells could keep above 95% of cell viability when incubated with 0-0.125 mg/mL of the prepared nanotheranostic agent. Thus, the prepared UCNPs@SiO_2_/HA/MB/ICG@PEG-TPP nanotheranostic agent showed negligible cytotoxicity and excellent applicability as the nanotheranostic agent.

Figure S5: Cell viability (%) under different concentrations of the UCNPs@SiO_2_/HA/MB/ICG@PEG-TPP nanotheranostic agent. Each concentration was operated five times and error bars represent standard deviation (n=5).

1. ***In vitro* ROSs detection**

The commerical fluorescent dye, 2,7-dichlorofluorescin diacetate (DCFH-DA), was used to investigate the ability of the prepared nanotheranostic agent to produce ROSs in living cells. As a cell-permeable oxidant-sensing probe, DCFH-DA would be converted into DCFH by related esterase when diffused into cells and then oxidized to DCF by ROSs with bright green fluorescence when excited (Hou et al., 2016; Kim et al., 2014). The green fluorescence could be recorded and analyzed with the flow cytometry. As demonstrated in Figure S6, MCF-7 cancer cells did not display obvious increased green fluorescence when only irradiated under NIR laser or only incubated with the nanoprobe, proving the applicability of the used NIR laser for PDT and negligible cytotoxicity of the prepared nanotheranostic agent. By contrast, when MCF-7 cancer cells were incubated with the prepared nanotheranostic agent and then irradiated under the NIR laser, there were significant increase in the fluorescence intensity of DCF.


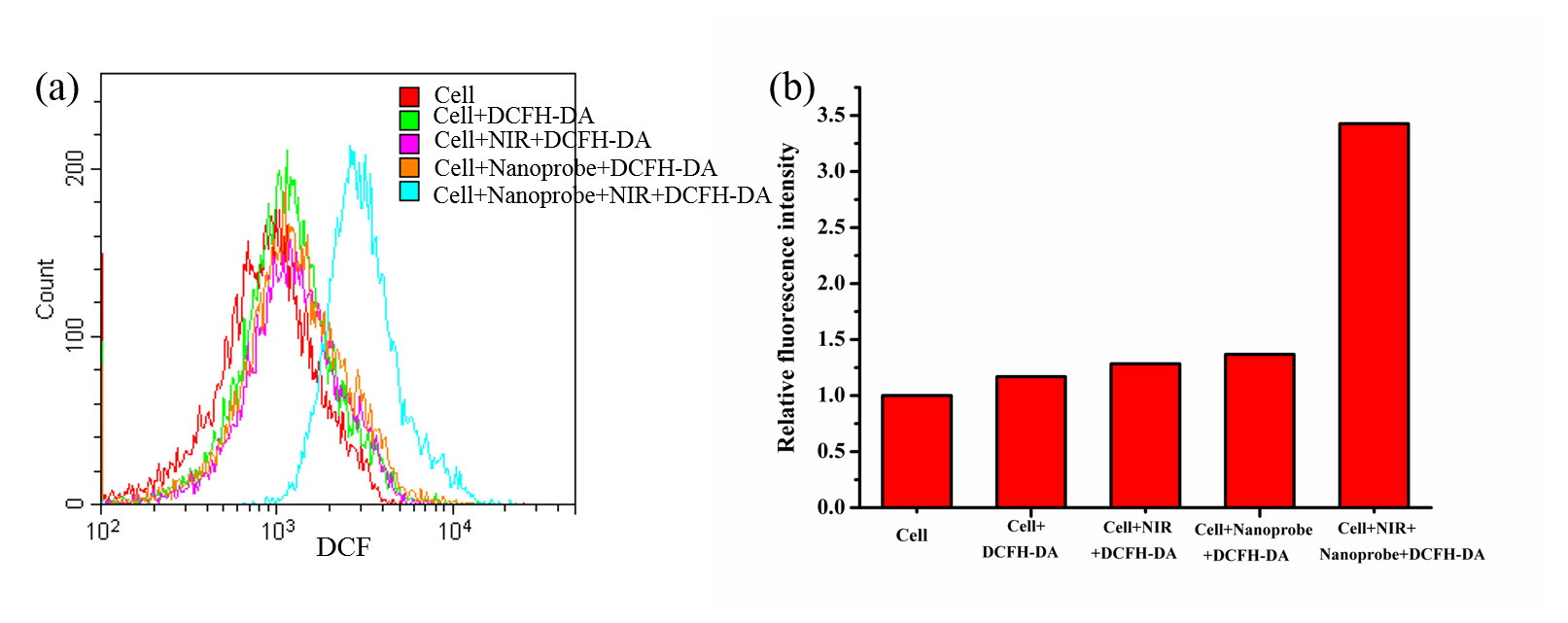


Figure S6: (a) flow cytometry analysis and (b) relative DCF fluorescence intensity of cancer cells with different treatments.

1. **PDT efficacy assay in living cells**

In this experiment, the PDT efficacy of the prepared nanotheranostic agent in living cells was first evaluated with the CCK-8 assay. MCF-7 cancer cells were treated with (a) PBS, (b) laser irradiation (980 nm, 1.5 W/cm^2^, 4.0 min with an interval of 1.0 min), (c) the prepared nanotheranostic agent only, (d) the prepared nanotheranostic agent and laser irradiation. To compare and investigate the PDT efficacy of the prepared nanotheranostic agent, three corresponding nanoprobes were prepared and used. Nanoprobe 1: UCNPs@SiO_2_/HA@PEG-TPP; Nanoprobe 2: UCNPs@SiO_2_/MB@PEG-TPP; Nanoprobe 3: UCNPs@SiO_2_/ICG@PEG-TPP; Nanoprobe 4: UCNPs@SiO_2_/HA/MB/ICG@PEG-TPP. The CCK-8 assay was operated according to the procedure in experimental section 2.4.5. As shown in Figure S7, the NIR irradiation or the nanoprobe alone would have no significant influence on the cell viability. However, when treated with the designed nanoprobe and then irradiated with the NIR laser, MCF-7 cancer cells showed great decrease in their viability. Moreover, MCF-7 cancer cells could only retain 17.3% of cell viability when treated with the designed UCNPs@SiO_2_/HA/MB/ICG@PEG-TPP nanotheranostic agent since the introduced triple PS molecules and designed photosensitizers-modulated sensitizing switch would increase the generation of ROSs and bring higher PDT efficacy.


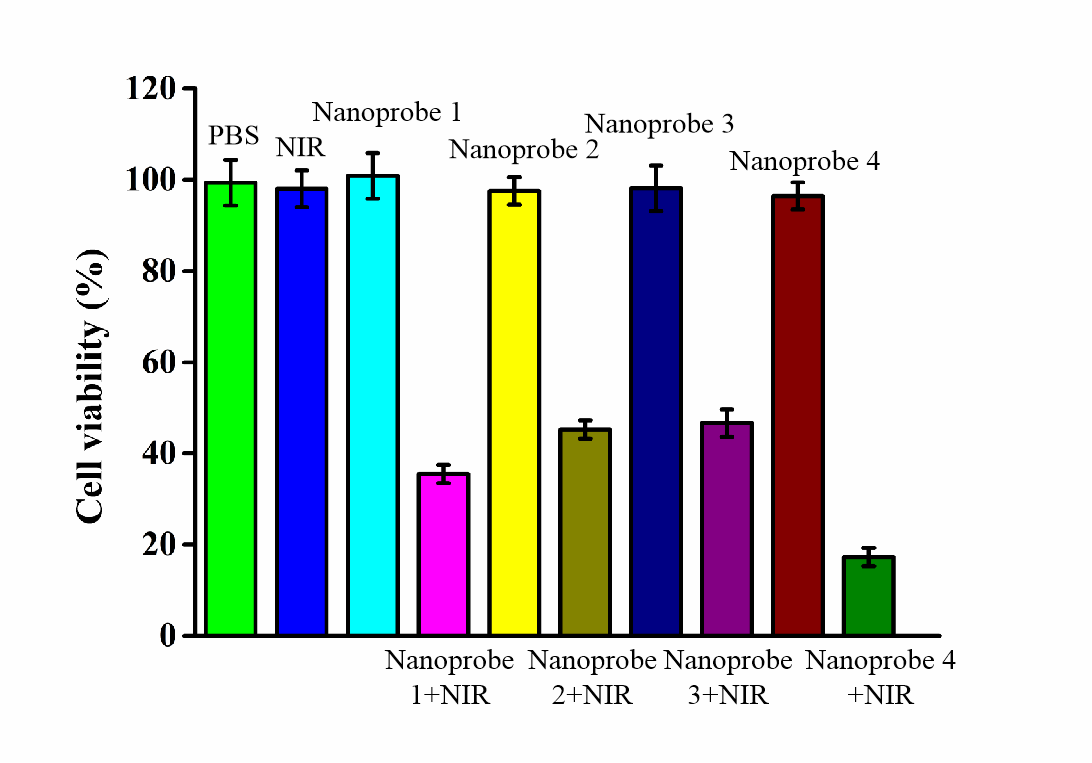


Figure S7: CCK-8 assay of MCF-7 cancer cells with different treatments. Control groups: PBS, NIR alone, Nanoprobe alone. Experiment groups: nanoprobe with NIR laser. Nanoprobe 1: UCNPs@SiO_2_/HA@PEG-TPP; Nanoprobe 2: UCNPs@SiO_2_/MB@PEG-TPP; Nanoprobe 3: UCNPs@SiO_2_/ICG@PEG-TPP; Nanoprobe 4: UCNPs@SiO_2_/HA/MB/ICG@PEG-TPP. Each treatment was operated five times and error bars represent standard deviation (n=5).

1. **H&E staining analysis**

On the fourteenth day, the mouse was sacrificed to obtain the main organs for hematoxylin and eosin (H&E) staining to investigate the biological toxicity of the prepared nanotheranostic agent. As shown in Figure S8, there was no obvious tissue abnormalities in the obtained main organs of mouse.


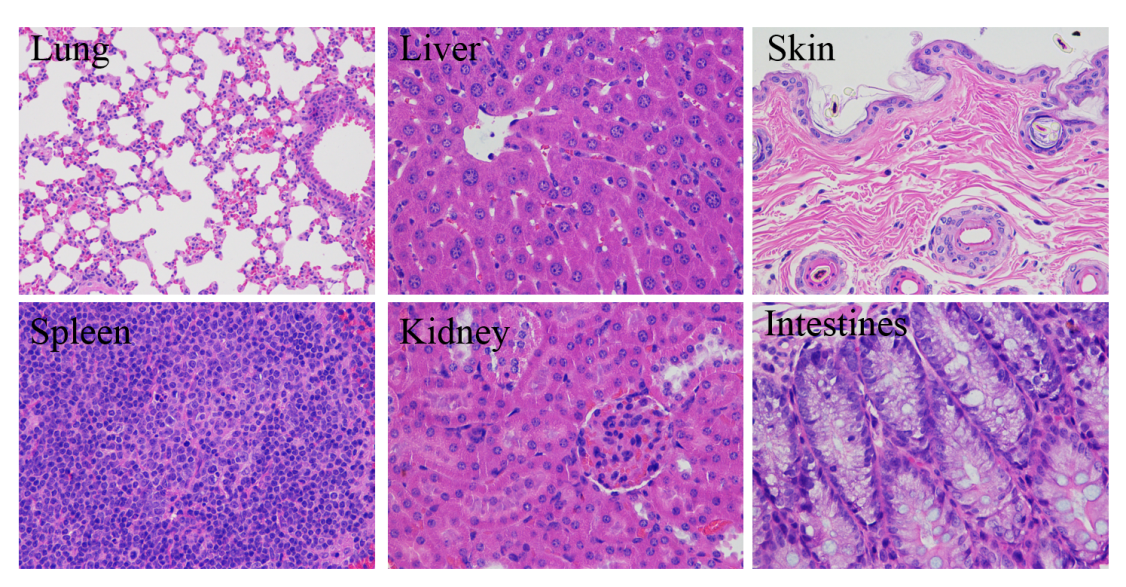


Figure S8: Bio-toxicity investigation of the prepared UCNPs@SiO_2_/HA/MB/ICG@PEG-TPP nanotheranostic agent to the main organs.

1. **Animal experiments**

In the animal experiments, four groups of mice were designed: (a) only injected with PBS, (b) laser irradiation alone, (c) only injected with the nanotheranostic agent, (d) injected with the nanotheranostic agent and then irradiated the tumor section after 12 h. The tumor volume (V=length×width^2^/2) was recorded every two days over a period for 13 days. As demonstrated in Figure S9, tumors in groups a-c showed significant increase while the tumor in group d showed obvious decrease on the thirteenth day.


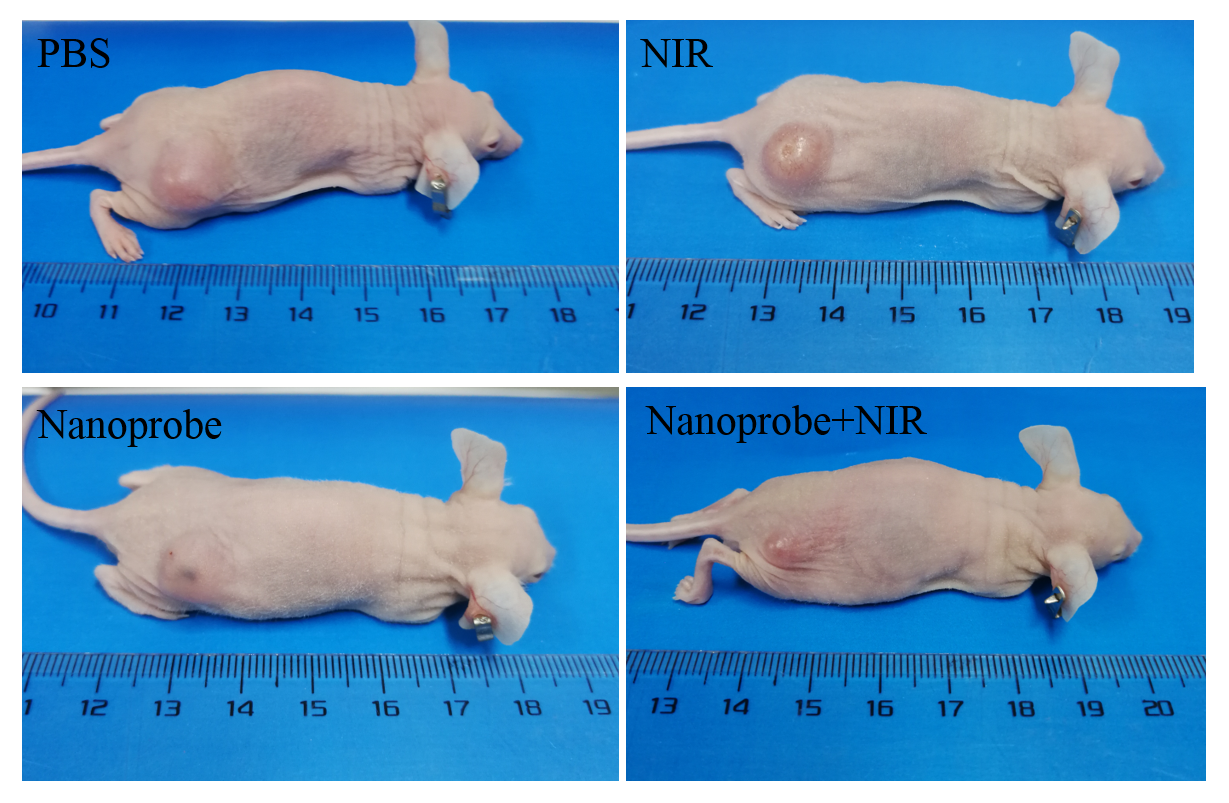


Figure S9: Photographs of tumor-bearing mice with different treatments on the thirteenth day.

**Reference**

1. Liu, B., Li, C. X., Yang, D. M., Hou, Z. Y., Ma, P. A., Cheng, Z. Y., et al. (*2014*). [Upconversion-luminescent core/mesoporous-silica-shell structured beta-NaYF](http://apps.webofknowledge.com.ezproxy.insead.edu/full_record.do?product=WOS&search_mode=GeneralSearch&qid=96&SID=E6rHe6naalawMruLN3D&page=1&doc=5)_[4](http://apps.webofknowledge.com.ezproxy.insead.edu/full_record.do?product=WOS&search_mode=GeneralSearch&qid=96&SID=E6rHe6naalawMruLN3D&page=1&doc=5)_[:Yb](http://apps.webofknowledge.com.ezproxy.insead.edu/full_record.do?product=WOS&search_mode=GeneralSearch&qid=96&SID=E6rHe6naalawMruLN3D&page=1&doc=5)^[3+](http://apps.webofknowledge.com.ezproxy.insead.edu/full_record.do?product=WOS&search_mode=GeneralSearch&qid=96&SID=E6rHe6naalawMruLN3D&page=1&doc=5)^[,Er](http://apps.webofknowledge.com.ezproxy.insead.edu/full_record.do?product=WOS&search_mode=GeneralSearch&qid=96&SID=E6rHe6naalawMruLN3D&page=1&doc=5)^[3+](http://apps.webofknowledge.com.ezproxy.insead.edu/full_record.do?product=WOS&search_mode=GeneralSearch&qid=96&SID=E6rHe6naalawMruLN3D&page=1&doc=5)^[@SiO](http://apps.webofknowledge.com.ezproxy.insead.edu/full_record.do?product=WOS&search_mode=GeneralSearch&qid=96&SID=E6rHe6naalawMruLN3D&page=1&doc=5)_[2](http://apps.webofknowledge.com.ezproxy.insead.edu/full_record.do?product=WOS&search_mode=GeneralSearch&qid=96&SID=E6rHe6naalawMruLN3D&page=1&doc=5)_[@mSiO](http://apps.webofknowledge.com.ezproxy.insead.edu/full_record.do?product=WOS&search_mode=GeneralSearch&qid=96&SID=E6rHe6naalawMruLN3D&page=1&doc=5)_[2](http://apps.webofknowledge.com.ezproxy.insead.edu/full_record.do?product=WOS&search_mode=GeneralSearch&qid=96&SID=E6rHe6naalawMruLN3D&page=1&doc=5)_ [composite nanospheres: fabrication and drug-storage/release properties](http://apps.webofknowledge.com.ezproxy.insead.edu/full_record.do?product=WOS&search_mode=GeneralSearch&qid=96&SID=E6rHe6naalawMruLN3D&page=1&doc=5). *Eur. J. Inorg. Chem.* 11, 1906-1913. doi: *10.1002/ejic.201301460*
2. Yan, F., Wu, H., Liu, H. M., Deng, Z. T., Liu, H., Duan, W. L., et al. (*2016*). Molecular imaging-guided photothermal/photodynamic therapy against tumor by iRGD-modified indocyanine green nanoparticles. *J. Control. Release* 224, 217-228. doi: *10.1016/j.jconrel.2015.12.050*
3. Hou, Z. Y., Deng, K. R., Li, C. X., Deng, X. R., Lian, H. Z., Cheng, Z. Y., et al. (*2016*). 808 nm Light-triggered and hyaluronic acid-targeted dual-photosensitizers nanoplatform by fully utilizing Nd^3+^-sensitized upconversion emission with enhanced anti-tumor efficacy. *Biomaterials* 101, 32-46. doi: *10.1016/j.biomaterials.2016.05.024*
4. Kim, J., Santos, Q. A., and Park, J. H. (*2014*). Selective photosensitizer delivery into plasma membrane for effective photodynamic therapy. *J. Control. Release* 191, 98-104. doi: *[10.1016/j.jconrel.2014.05.049](https://doi.org/10.1016/j.jconrel.2014.05.049)*
5. Wei, W., Chen, G., Baev, A., He, G. S., Shao, W., Damasco, J., et al. (*2016*). Alleviating luminescence concentration quenching in upconversion nanoparticles through organic dye sensitization. *J. Am. Chem. Soc.* 138, 15130-15133. doi: *10.1021/jacs.6b09474*
